# Identification of a Novel Function of CX-4945 as a Splicing Regulator

**DOI:** 10.1371/journal.pone.0094978

**Published:** 2014-04-17

**Authors:** Hyeongki Kim, Kwangman Choi, Hyunju Kang, So-Young Lee, Seung-Wook Chi, Min-Sung Lee, Jaehyoung Song, Donghwa Im, Yura Choi, Sungchan Cho

**Affiliations:** 1 Targeted Medicine Research Center, Korea Research Institute of Bioscience and Biotechnology, Cheongwon, Chungbuk, Korea; 2 Department of Biomolecular Science, University of Science and Technology, Daejeon, Korea; 3 International Cooperation Office, Ministry of Food & Drug Safety, Cheongwon, Chungbuk, Korea; 4 BioMedical Proteomics Research Center, Korea Research Institute of Bioscience and Biotechnology, Cheongwon, Chungbuk, Korea; Florida Atlantic University, United States of America

## Abstract

Alternative splicing is a nearly ubiquitous versatile process that controls gene expression and creates numerous protein isoforms with different functions from a single gene. The significance of alternative splicing has been confirmed by the increasing number of human diseases that are caused by misregulation of splicing events. Very few compounds, however, have been reported to act as inhibitors of alternative splicing, and their potential clinical use needs to be evaluated. Here, we report that CX-4945, a previously well-characterized inhibitor of casein kinase 2 (CK2) and a molecule currently in clinical trials (Phase II) for cancer treatment, regulates splicing in mammalian cells in a CK2-independent manner. Transcriptome-wide analysis using exon array also showed a widespread alteration in alternative splicing of numerous genes. We found that CX-4945 potently inhibits the Cdc2-like kinases (Clks) *in vitro* and in turn, leads to suppression of the phosphorylation of serine/arginine-rich (SR) proteins in mammalian cells. Surprisingly, the overall efficacy of CX-4945 on Clks (IC_50_ = 3–90 nM) was stronger than that of TG-003, the strongest inhibitor reported to date. Of the Clks, Clk2 was most strongly inhibited by CX-4945 in an ATP-competitive manner. Our research revealed an unexpected activity of the drug candidate CX-4945 as a potent splicing modulator and also suggested a potential application for therapy of diseases caused by abnormal splicing.

## Introduction

The removal of introns and rejoining of adjacent exons from nascent transcripts by the process of pre-mRNA splicing is an essential step in eukaryotic gene expression [1]. Most pre-mRNAs in higher eukaryotes can be spliced in several different ways to produce multiple mRNAs in a process called alternative splicing, allowing a single gene sequence to be expressed as numerous protein isoforms with different functions [2]. In this way, alternative splicing contributes to the cellular complexity and generates the phenotypic diversity of higher eukaryotes without the need to expand the genome [3]. Global analysis of the human transcriptome estimates that up to 95% of multiple intron-containing genes undergo alternative splicing [4], [5]. Importantly, alternative splicing is elaborately regulated in a tissue-, developmental stage- and signal-dependent manner. Aberrations in splicing due to mutations in pre-mRNAs or splicing machinery have been increasingly found to be associated with a wide range of human diseases, such as cancers, neurodegenerative diseases, viral diseases, and autoimmune diseases [3], [6]–[9].

Alternative splicing is highly regulated by the elaborate and complex interplay of *trans*-acting splicing factors and *cis*-acting pre-mRNA elements [1], [10]. Particularly, the serine/arginine-rich (SR) proteins, which are one of the *trans*-acting splicing factors, play an essential role in alternative as well as constitutive splicing [11], [12]. SR proteins are composed of one or two RNA recognition motifs at the N-terminus and an arginine/serine dipeptide repeat (RS) domain at the C-terminus [13], [14]. More importantly, phosphorylation of SR proteins has been demonstrated to be crucial for the regulation of splicing through alterations in protein-protein and protein-RNA interactions [15] as well as in subcellular localization [16], [17]. Several kinases that phosphorylate SR proteins have been identified: the Cdc2-like kinases (Clks) including Clk1, Clk2, Clk3, and Clk4 [18]–[20] and the SRPK family kinases (SRPK1 and SRPK2) [21], [22]. These kinases have been considered attractive targets for pharmacological modulation of alternative splicing, and such modulation is useful for understanding the splicing mechanism as well as developing drugs for treatment of splicing-related diseases. Only a small number of constitutive or alternative splicing inhibitors, particularly ones targeting Clks and SRPKs, however, have been identified [23]–[30].

Here, we unexpectedly identified a new function of CX-4945, a potent and selective inhibitor of casein kinase 2 (CK2) currently in clinical trials for cancer treatment [31], as a potent splicing modulator. The splicing regulatory function of CX-4945 was not dependent on its previously well-characterized CK2 inhibition. Importantly, we demonstrated that CX-4945 regulates alternative splicing via modulation of SR phosphorylation by potently targeting Clks in an ATP-competitive manner. These findings suggest a new therapeutic application of CX-4945 for diseases caused by abnormal splicing.

## Materials and Methods

### Cell culture, drug treatment, and transfection

Cells (293T, Huh7, and HepG2) were cultured in Dulbecco's modified Eagle's medium (DMEM) containing 10% fetal bovine serum (FBS; Hyclone) supplemented with 1% penicillin and streptomycin. The CK2 inhibitors CX-4945 (Selleck), TBB (Sigma), TBCA (Calbiochem), and Clk inhibitor TG-003 (Sigma) were dissolved in DMSO before incubation with the cells. The 293T cells were transfected in 6-well plates with siRNAs (Bioneer) at a final concentration of 100 nM using Lipofectamine RNAiMax (Invitrogen) according to the manufacturer's instructions.

### RNA extraction, RT-PCR, and quantitative real-time PCR

To analyze the effect of compounds on pre-mRNA splicing, 293T, Huh7, and HepG2 cells were treated with the indicated concentrations. Total RNAs were extracted using the TRIzol reagent (Invitrogen) followed by cDNA synthesis using the Omniscript RT kit (Qiagen) and an oligo-dT primer. PCR was performed at 95°C for 30 s, 55°C for 30 s, and 72°C for 1 min for 35 cycles using GoTaq Green Master Mix (Promega). Quantitative real-time PCR analysis was performed using the BioRad IQ SYBR Green Supermix. All gene expression experiments were performed independently at least twice. All primers are listed in Table S1 in File S1.

### Quantitative western blot analysis

Total cell extracts were prepared, resolved on SDS-PAGE, and transferred to a PVDF membrane (GE Healthcare). Proteins that reacted with antibodies were detected on the membrane using a WEST-ZOL Plus western blotting detection system (Intron Biotechnology), analyzed subsequently with an LAS-4000 image analyzer (Fujifilm, Tokyo, Japan), and then quantified using image analysis software (ImageJ, NIH, USA). Anti- SRSF1, -SRSF4, -SRSF9, -GAPDH, -CK2 α, and -CK2 α′ antibodies were obtained from Santa Cruz biotechnology. Anti-phosphoSR protein (1H4) antibody was obtained from Millipore, anti-AKT antibody was obtained from Cell Signaling, and anti-pAKT(S129) antibody was purchased from Abcam. Anti-hnRNP A1 antibody was kindly provided by Gideon Dreyfuss (University of Pennsylvania). All experiments were performed independently at least twice.

### *In vitro* kinase assays

The kinase assays were conducted using the Kinase Profiler services offered by Millipore and Life Technologies, which utilize a radiometric filter-binding assay and fluorescence-based immunoassay, respectively. Detailed protocols of the kinase assays conducted by Millipore and Life Technologies can be found at http://www.millipore.com/techpublications/tech1/pf3036 and http://www.lifetechnologies.com/kr/ko/home/products-and-services/services/custom-services/screening-and-profiling-services/selectscreen-profiling-service/selectscreen-kinase-profiling-service, respectively. Briefly, for *in vitro* kinase assay by Millipore, recombinant kinases were incubated with 8 mM MOPS (pH 7.0), 0.2 mM EDTA, 20∼250 µM a synthetic SR-rich substrate, 10 mM magnesium acetate, and γ-^33^P-ATP. The reaction was initiated by the addition of magnesium/ATP. After incubation for 40 minutes at room temperature, the reaction was stopped by the addition of 3% phosphoric acid solution. 10 µL of the reaction was then spotted onto a P30 filtermat and washed three times for 5 minutes in 75 mM phosphoric acid and once in methanol prior to drying and scintillation counting. For *in vitro* kinase assay by Life Technologies, recombinant kinases were incubated with 50 mM HEPES (pH 7.5), 0.01% BRIJ-35, 10 mM MgCl_2_, 1 mM EGTA, and Ser/Thr peptide. After the 1 hour kinase reaction, 5 µL of a 1∶512 dilution of Development Reagent solution was added. The reaction was developed and terminated, and then the fluorescence ratio was calculated according to the manufacturer's protocol.

The inhibitory activities for each kinase (Clk1, Clk2, Clk3, Clk4, SRPK1, SRPK2, and CK2) were measured with 5 concentrations of CX-4945 over a range of 0.001 to 10 µM, and IC_50_ values were determined using the GraphPad Prism 5 software. To determine whether CX-4945 acts by competing with ATP for inhibition of Clk2, kinase activity was measured in the presence of various concentrations of ATP (5, 15, 45, and 135 µM), and the IC_50_ values were determined using the GraphPad Prism 5 software. All experiments were performed twice.

### Affymetrix exon array and statistical analysis

The 293T cells were incubated in the presence or absence of 10 µM CX-4945 for 12 hours, and total RNAs were purified using the TRIzol reagent. The fragmented and end-labeled single-stranded cDNAs were prepared and hybridized to Affymetrix-GeneChip Human Exon 1.0 ST arrays (Affymetrix). Affymetrix Expression Console Software was used to perform quality assessment. Affymetrix exon array data was analyzed using GeneSpring 12.6 inclusive of GX (Agilent Technologies). Three independent experimental samples were examined.

### Computer-aided molecular docking

To build a structural model of human Clk2 in complex with CX-4945, we performed molecular docking studies with the LigandFit module in Discovery Studio 2.5 software (Accelrys) [32]. The CX-4945 ligand was generated by ChemBioDraw, and the energy-minimized structure was transferred to Discovery Studio 2.5. The crystal structure of Clk2 obtained from the RCSB Protein Data Bank (PDB code: 3NR9) was used for docking studies. The number of generated poses was set to 50 for the ligand, and default settings were used for the other parameters. Scoring function scores were obtained with Dock score, Ligscore1, Ligscore2, PLP1 score, PMF score, and Jain score. The structure with the highest Dock score was selected as a final structural model. Figures of the complex were drawn using the PyMOL software package.

## Results

### Effects of CX-4945 on pre-mRNA splicing

**Figure 1:**
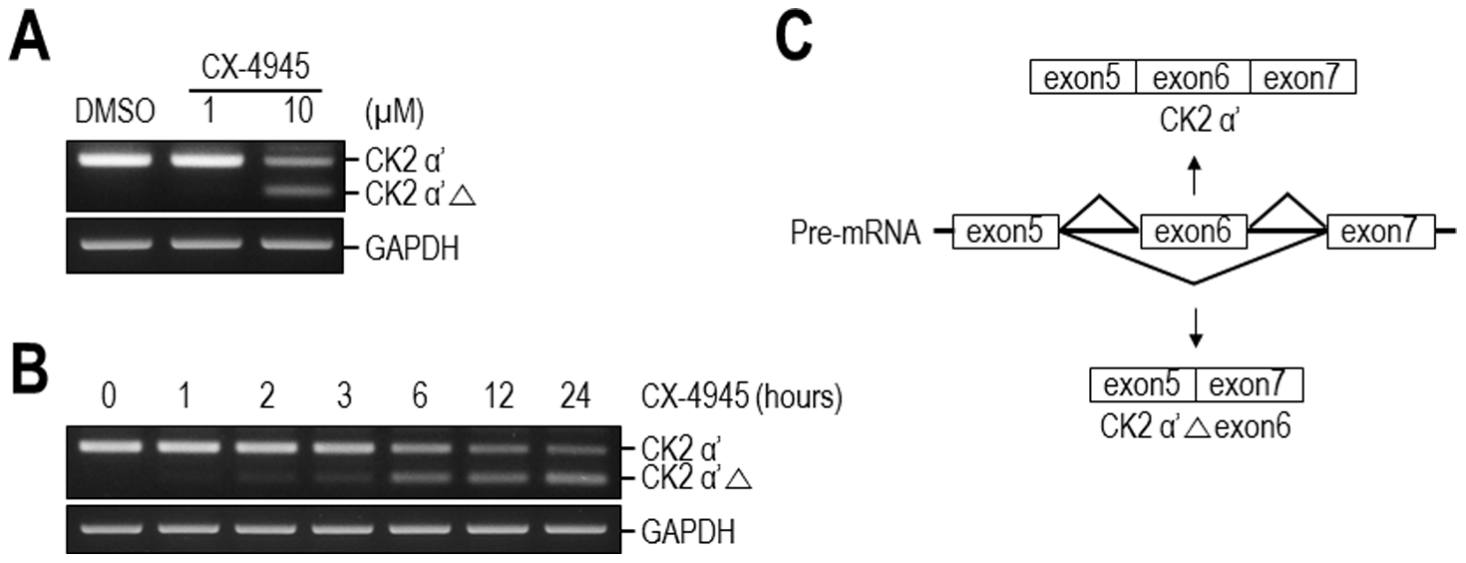
Effect of CX-4945 on pre-mRNA splicing. (A) CX-4945 induces expression of an unexpected CK2 α′ transcript, CK2 α′△. RT-PCR corresponding to exons 5–6–7 was performed with total RNA from DMSO- or CX-4945-treated 293T cells. (B) CX-4945 rapidly induces the expression of CK2 α′△. 293T cells were treated with CX-4945 for the times indicated prior to RNA preparation and RT-PCR analysis. GAPDH mRNA was analyzed as a control. (C) Analysis of the DNA sequence of CK2 α′△ revealed that it represents the exon6-skipped product (CK2 α′△exon6) of CK2 α′ pre-mRNA. The normally spliced (CK2 α′) and alternatively spliced (CK2 α′△exon6) products are illustrated schematically.

CK2, a ubiquitously expressed and pleiotropic serine/threonine kinase, forms a tetrameric holoenzyme composed of two catalytic alpha and/or alpha' (α and/or α′) subunits and two regulatory beta (β) subunits [33]. The expression of CK2 is abnormally elevated in a wide variety of cancers [34]–[37]. The role of CK2 in cancer has recently been described, and its pharmacologic inhibition is considered to be a potential therapeutic option for several cancers. In our preliminary experiments that investigated a possible role of CK2 in other physiologies including lipid metabolism, we occasionally observed that CX-4945, a well-characterized potent inhibitor of CK2, induced alterations in the splicing pattern of CK2 α′ pre-mRNA. To examine this further, 293T cells were treated with 1 µM and 10 µM CX-4945 for 12 hours, and the expression of CK2 α′ mRNA was analyzed by RT-PCR (Figure 1A). Intriguingly, the amount of PCR product corresponding to normal CK2 α′ was reduced in cells treated with 10 µM CX-4945 compared with that in cells treated with DMSO. In addition, we observed a concomitant appearance of another PCR product (CK2 α′△), which was slightly smaller than the normal CK2 α′ product. Even though the amount of normal CK2 α′ mRNA decreased after CX-4945 treatment, the total amount of normal CK2 α′ and the smaller-sized CK2 α′ mRNA was not altered (Figure S1 in File S1). Moreover, this inverse correlation between normal CK2 α′ and the new product became more obvious with longer treatment with CX-4945, and the appearance of a new product occurred rapidly, as early as 2 or 3 hours following CX-4945 exposure (Figure 1B). Sequence analysis of this new product revealed that it was exon6-skipped (CK2 α′ △exon6), an alternatively spliced form of CK2 α′ pre-mRNA (Figure 1C). CK2 α′ △exon6 mRNA has not been reported previously, and this mRNA hypothetically encodes an in-frame CK2 α′ protein with a deleted exon6. In fact, CK2 α′ △exon6 protein was barely detectable in several cultured cell types even after CX-4945 treatment. These results demonstrate that CX-4945 induces an abnormal alternative splicing of CK2 α′ pre-mRNA and also suggest that CX-4945 has an effect on pre-mRNA splicing.

### Transcriptome-wide analysis of the effects of CX-4945 on pre-mRNA splicing

**Figure 2:**
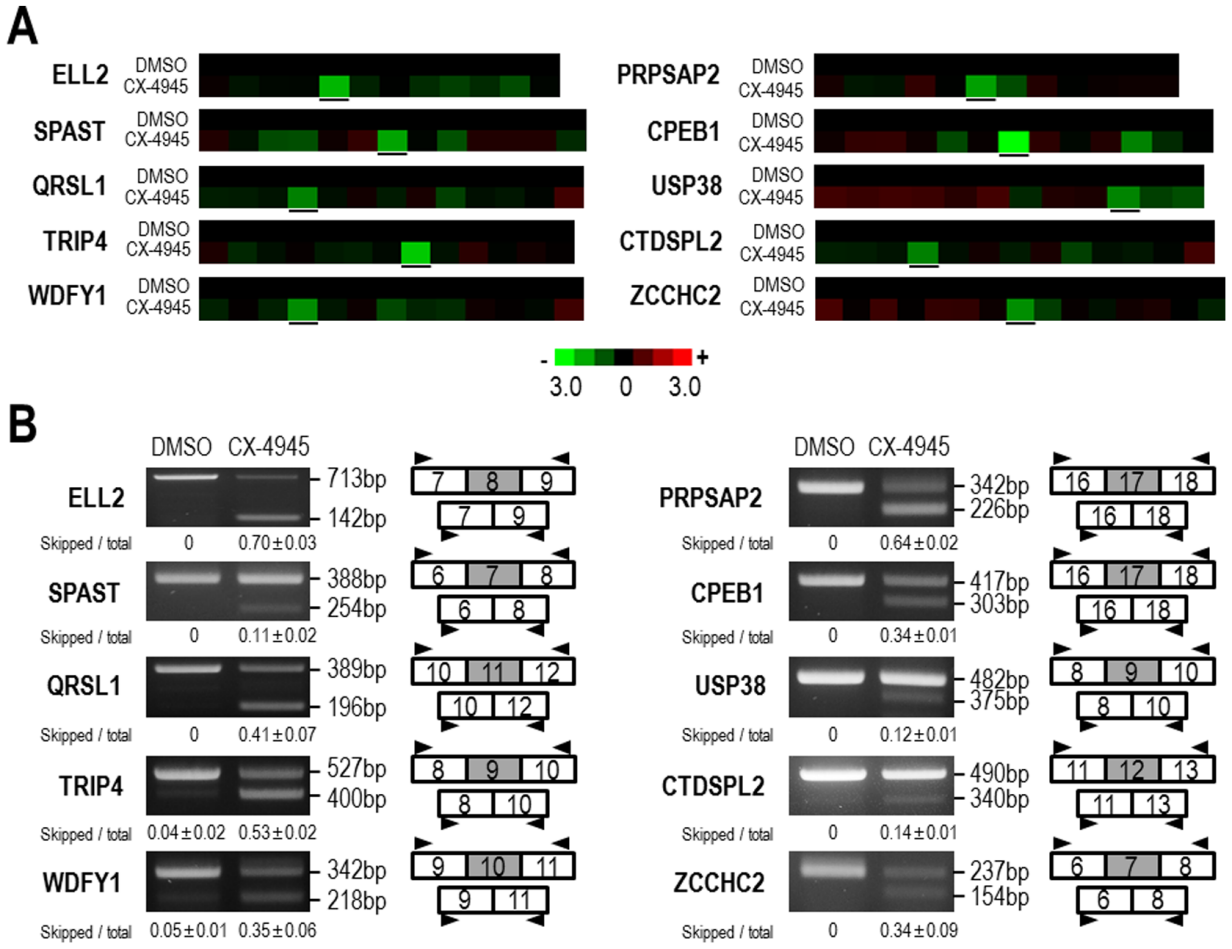
The widespread effect of CX-4945 on alternative splicing of numerous genes. (A) 293T cells were treated with DMSO or 10 µM CX-4945 for 12 hours, and total RNA was prepared. The cDNA was synthesized and further processed for exon array as described in Materials and Methods. All experiments were performed three times, and representative heat maps of selected transcripts from the exon arrays are shown. Selected exons for each mRNA are highlighted with black underlines. (B) RT-PCR analysis was performed for the ELL2, SPAST, QRSL1, PRPSAP2, CPEB1, USP38, TRIP4, CTDSPL2, WDFY1 and ZCCHC2 genes with the same RNA used for the exon arrays. The exonic structure of the RT-PCR products and the exonic location of PCR primers are indicated with tandem rectangles and arrowheads, respectively. PCR products were quantified using ImageJ software, and the ratios of skipped product (skipped/total PCR product) were calculated. The average and SD values were determined from two independent experiments and are presented below the PCR data.

The effect of CX-4945 on splicing of CK2 α′ pre-mRNA prompted us to examine its effect on splicing at a transcriptome-wide level. Therefore, total RNA purified from 293T cells that had been treated with DMSO or CX-4945 (10 µM) were analyzed by exon array. Transcriptome analysis with the Affymetrix GeneChip Human Exon 1.0 ST Array, which contains multiple probes per exon, allowed us to search for variations at the exon level [38]. Treatment with CX-4945 had a profound effect on alternative splicing in 293T cells. A notable proportion of exons (∼4.67%, 8,968 out of 191,871 exons) were affected by more than 4-fold, but only 0.44% of genes (82 out of 18,673 genes) were affected at the whole-transcript level, indicating a preferential effect of CX-4945 on alternative splicing regulation. Further analysis characterized the 8,968 affected exons into 1,555 included exons and 7,413 skipped exons. To validate this observation, we randomly chose 15 exons from those affected by more than 2-fold and examined alterations in alternative splicing using RT-PCR of the same total RNAs used in the exon array experiments. Representative mRNAs containing selected exons are illustrated by heat maps, and all of the selected exons were denoted by black underlines (Figure 2A). Even though other exons in the same mRNAs were also affected by CX-4945 treatment, the selected exons exhibited outstanding alterations that are depicted by bright green color (Figure 2A). Different exons are commonly affected differentially by a stimulus or splicing inhibition, depending on *cis*-acting elements on exon/intron and activities of *trans*-acting factors [1]. Out of 15 selected genes that had been predicted to be affected by treatment with CX-4945, ten were validated by RT-PCR (Figure 2B), while the other five exons showed little changes in splicing patterns. These alternatively spliced isoforms have not been reported previously, and thus, CX-4945 induces a wide-range of abnormal alternative splicing of numerous genes. Nevertheless, we also observed the alteration of normal alternative splicing of Bcl-X and RON pre-mRNAs (Figure S2A in File S1), which have been studied extensively with respect to cancer [39], [40]. Moreover, we observed changes in splicing of Clk1/Sty pre-mRNA (Figure S2B in File S1), and these alterations have been previously shown in response to TG-003 [41]. Together, these results demonstrate that CX-4945 has wide-ranging effects on normal and abnormal alternative splicing of numerous genes.

### CX-4945 affects alternative splicing in a CK2-independent manner

**Figure 3:**
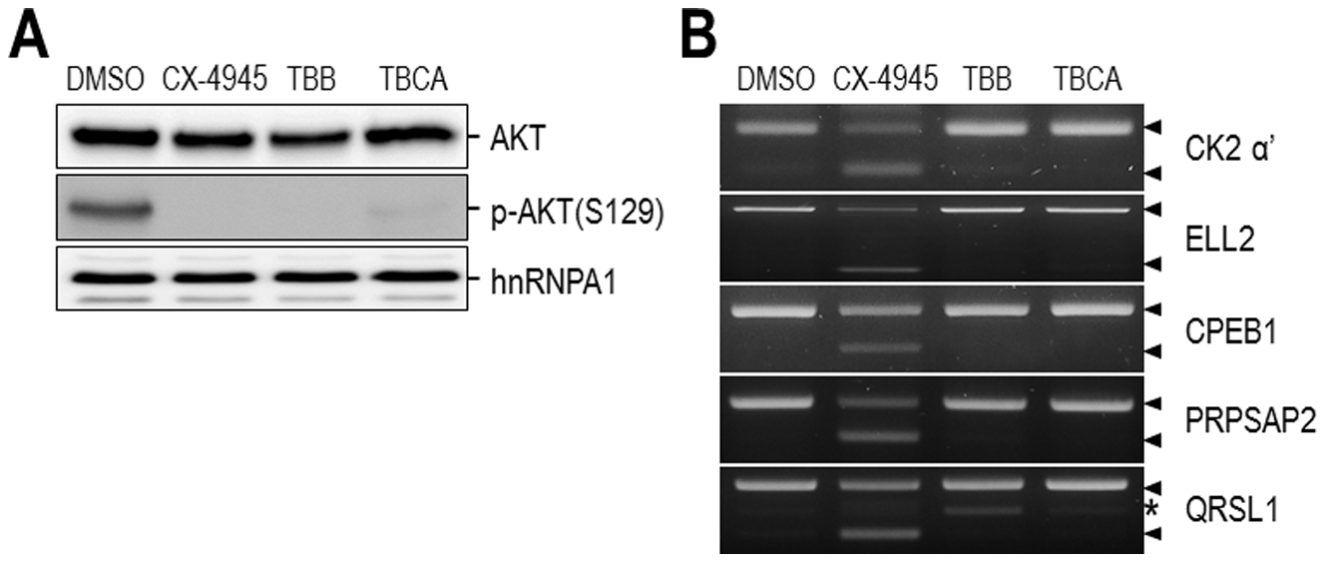
CX-4945 affects alternative splicing in a CK2-independent manner. (A) Two other inhibitors of CK2, TBB and TBCA, were also tested to determine whether splicing regulation by CX-4945 is related to CK2 activity. 293T cells were treated with 10 µM CX-4945 or 100 µM TBB or and TBCA for 12 hours. Inhibitory activities of CX-4945, TBB, and TBCA on CK2 were assessed by examination of phosphorylation of AKT(S129), the major substrate of CK2. Total levels of AKT were also monitored as a control. hnRNP A1 was used as a loading control. (B) Alternatively spliced CK2 α′, ELL2, CPEB1, PRPSAP2, and QRSL1 mRNAs were examined by RT-PCR. PCR products of normal and alternatively spliced isoforms are denoted by black arrowheads. An extra PCR product of the QRSL1 gene appeared in TBB-treated samples and is denoted by an asterisk. All experiments were performed twice, and representative data for each are presented.

CX-4945 is a potent and selective orally available small molecule inhibitor of CK2 and is in clinical trials for cancer treatment [31]. Therefore, in order to determine whether CK2 inhibition is responsible for the observed alterations in splicing caused by CX-4945, we utilized two other CK2 inhibitors, 4,5,6,7-tetrabromobenzotriazole (TBB) and tetrabromocinnamic acid (TBCA) [42], [43]. If the other inhibitors do not exert the same effect as CX-4945 on splicing, then we can exclude the possibility that splicing regulation by CX-4945 is mainly related to CK2 inhibition. Firstly, attenuation of PI3K/AKT signaling by CK2 inhibitors was assessed by examination of the dephosphorylation of AKT at the CK2-specific site (S129), as this attenuation has been well characterized to be dependent on CK2 [44]. CX-4945, TBB, and TBCA all efficiently blocked the CK2-mediated phosphorylation of AKT at S129 (Figure 3A), confirming the inhibitory activity of these compounds. Under the same conditions, the alternative splicing pattern of several genes that had been previously validated in Figure 2B were analyzed using RT-PCR. Surprisingly, CX-4945 induced dramatic changes in alternative splicing of all four genes, while TBCA and TBB had a negligible effect (Figure 3B). Only the treatment with TBB induced the appearance of an additional PCR product. Sequence analysis of this extra product revealed that it includes only the 3′ part of exon 11 of QRSL1 mRNA rather than the whole exon 11, and this product appears to be produced by cryptic splicing (Figure S3 in File S1). Considering the profound effect of CX-4945 on alternative splicing, this minor change induced by TBB may be an indirect effect rather than direct modulation of a splicing event. In addition to pharmacological inhibition, efficient siRNA-mediated knockdown of CK2 α and/or α′ did not alter the splicing pattern (Figure S4 in File S1). Collectively, these results demonstrate that splicing regulation by CX-4945 is not mainly related to its inhibition of CK2.

### CX-4945 modulates SR protein phosphorylation by directly inhibiting the Clks

**Figure 4:**
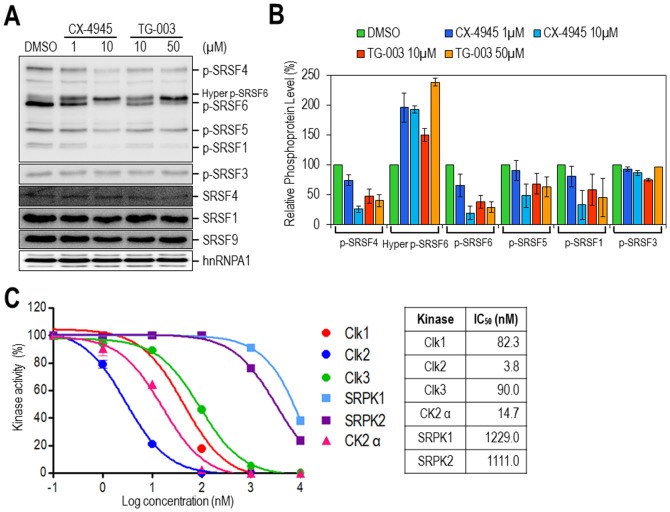
CX-4945 modulates SR protein phosphorylation via direct inhibition of Clks. (A) Total protein extracts from 293T cells treated with CX-4945 (1 and 10 µM) or TG-003 (10 and 50 µM) for 1 hour were separated by SDS-PAGE, and phosphorylated SR proteins were monitored by western blotting using the phosphoSR monoclonal antibody (1H4). SRSF1, SRSF4, SRSF9, and hnRNP A1 proteins were also monitored as controls. Western blotting was performed twice, and representative data are presented. (B) The phosphorylated SR proteins in (A) were quantified, and amounts of each protein relative to those of DMSO-treated samples are shown. The average and SD values were determined from two independent experiments. (C) The potent inhibition of Clks by CX-4945 was observed in *in vitro* kinase assays conducted by Life Technologies using recombinant human Clk1, Clk2, Clk3, SRPK1, SRPK2, and Ck2 α proteins. The average and SD values were determined from two independent assays. The IC_50_ values were determined using PRISM software. Detailed experimental procedures are described in the Materials and Methods.

The observation that splicing regulation by CX-4945 is independent of CK2 directed us to examine the potential effect of CX-4945 on SR protein phosphorylation, which is a major determinant of alternative splicing regulation. The phosphorylation of SR proteins can easily be monitored using an antibody (mAb1H4) that specifically recognizes the phospho-SR peptide on SR proteins [45]. Overall, treatment of 293T cells with CX-4945 induced profound changes in the phosphorylation status of SR proteins. Levels of phosphorylated SRSF4, SRSF6, SRSF5, and SRSF1 were considerably decreased. Intriguingly, the upper band of phospho-SRSF6 was dramatically increased with a concomitant decrease of the smaller phospho-SRSF6 band. Throughout our studies, the disappearance of phospho-SRSF6 was inversely correlated with the appearance of the upper band that has been speculated to represent hyperphosphorylated SRSF6 (40, 41) (Figure 4A and 4B). The effect on the phosphorylation of SR proteins was dose-dependent (compare the results from treatment with 1 and 10 µM CX-4945 in Figure 4A). Moreover, unlike CX-4945, TBB and TBCA did not induce the alteration of SR protein phosphorylation (Figure S5 in File S1), and this finding supports our conclusion that splicing regulation by CX-4945 is not dependent on CK2 inhibition. More importantly, the differential effects of CX-4945 on a series of SR proteins were almost identical to those observed for TG-003, a previously well-characterized SR protein phosphorylation regulator that acts by inhibiting Clks [26] (Figure 4A and 4B). Notably, the regulatory efficacy of CX-4945 on SR protein phosphorylation was stronger than that of TG-003, and a similar effect on SR protein phosphorylation was only achieved with a higher concentration of TG-003 (compare the results from 1 µM CX-4945 and 10 µM TG-003 in Figure 4A).

Considering the profound effect of CX-4945 on the phosphorylation states of SR proteins and their altered patterns in a manner similar to that of TG-003, we tested the effects of CX-4945 on two major classes of kinases, the Clk family (Clk1, Clk2, and Clk3) and SR protein kinases (SRPK1 and SRPK2), which target and phosphorylate SR proteins [18], [19], [21], [22]. Kinase assays using human recombinant kinases and an SR-rich peptide substrate as described in Materials and Methods revealed that CX-4945 potently inhibits the activity of Clks (Figure 4C). CX-4945 strongly inhibited all three Clks with an IC_50_ of 3–90 nM, while this compound only weakly inhibited SRPKs with an IC_50_ of >1,000 nM (Figure 4C). Surprisingly, the overall efficacies of CX-4945 on Clks were much stronger than those of TG-003, a commonly used potent Clk inhibitor (Figure S6 in File S1). Furthermore, Clk2 was the most strongly inhibited of the Clks by CX-4945 and had the lowest IC_50_ of 3.8 and 2.9 nM (Figure 4C and S6A, respectively), which is an unprecedented inhibitory profile for Clks. Most of the known Clk inhibitors including TG-003 have preferential efficacy on Clk1 and Clk4 over Clk2 and Clk3 by at least 5-fold [26]. To our knowledge, this is the first report of a chemical that exhibits preferential effects on Clk2. Moreover, the inhibitory efficacy of CX-4945 on Clk2 (IC_50_ of 3.8 nM) was even higher than or at least comparable to CX-4945 inhibition of CK2 (IC_50_ of 14.7 nM), the previously well-characterized target of CX-4945 (Figure 4C). Collectively, these results clearly demonstrate that CX-4945 targets Clks with high potency and also suggests that direct inhibition of Clks by CX-4945 most likely causes the altered phosphorylation of SR proteins in cells.

### CX-4945 inhibits Clk2 in an ATP-competitive way

**Figure 5:**
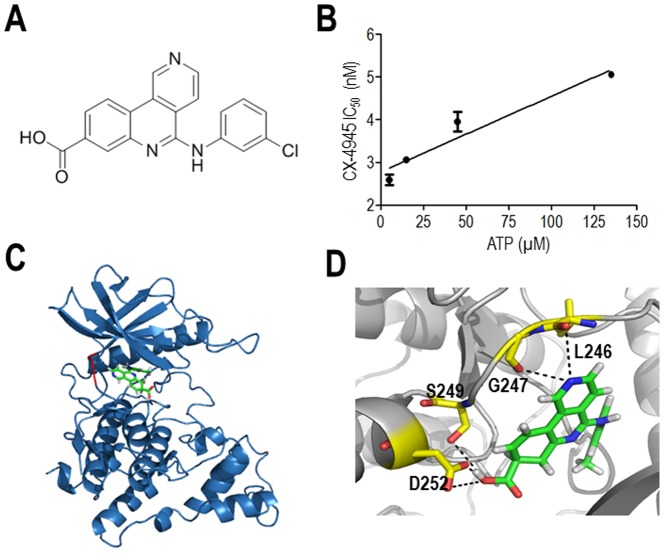
ATP-competitive inhibition of Clks by CX-4945. (A) Diagram of the chemical structure of CX-4945 (B) Competition of CX-4945 with ATP for inhibition of Clk2 (C) Structural model of the Clk2 in complex with CX-4945. CX-4945 was predicted to bind to the ATP-binding site of Clk2. (D) The predicted binding mode between CX-4945 and Clk2. Amino acids in the active site are denoted by yellow sticks, and CX-4945 is indicated by green sticks. Hydrogen bonds are denoted as dotted black lines, and oxygen and nitrogen atoms are indicated as red and blue sticks, respectively.

Previously, CX-4945 was found to inhibit CK2 activity by binding to the ATP-binding pocket and competing with ATP [46]. Therefore, to gain a better understanding of the inhibitory mechanism of CX-4945, we chose Clk2, the most strongly affected kinase, and measured the kinase activity with varying concentrations of ATP in the presence of different concentrations of CX-4945 *in vitro*. Biochemical kinase assays with increasing ATP concentrations revealed that CX-4945 inhibits Clk2 in an ATP-competitive way as expected (Figure 5B). Moreover, we performed molecular docking studies using the Ligand-Fit module program with Discovery Studio to gain insight into the orientation of CX-4945 in the ATP-binding pocket of Clk2 and the interaction between Clk2 and CX-4945 at the molecular level. Computer-aided molecular docking revealed that CX-4945 fits well within the ATP-binding pocket, and four hydrogen bonds were predicted to exist between the inhibitor and adjacent residues (L246, G247, S249, and D252) in the ATP-binding pocket of Clk2 (Figure 5C and 5D). In particular, the hydrogen bond between the hydroxyl group of CX-4945 and D252 on Clk2 seems to be crucial for this binding based on the fact that aspartate in the ATP-binding pocket of CK2 is similarly involved in CX-4945 binding [46] (Figure 5A and 5D). To more precisely understand the molecular basis of inhibition, a co-crystal structure of CX-4945 and Clk2 should be determined in the future. Overall, these results strongly support the notion that direct inhibition of Clk2 by CX-4945 occurs via ATP competition.

## Discussion

Numerous human diseases including cancer, viral infections, inflammatory responses, and neurological defects are caused by misregulation of splicing events [8], [47]–[49]. Identification of small molecules capable of correcting and/or inhibiting pathological splicing events is, therefore, important for future therapeutic approaches. In the present study, we show that CX-4945, the potent and selective CK2 inhibitor that is currently in clinical trials for cancer treatment, has a profound effect on alternative splicing. The new function of CX-4945 as a splicing modulator was identified firstly by the observation of an additional unexpected isoform of CK2 α′ mRNA in response to CX-4945 treatment of 293T cells (Figure 1A). Thereafter, a widespread effect on alternative splicing of numerous transcripts was found using exon array analysis, and some of these alterations were confirmed with RT-PCR (Figure 2A and 2B). It is notable that the effect of CX-4945 on alternative splicing was not restricted to either inclusion or exclusion of exons, even though exon exclusion was more prevalent. Moreover, many of the alternatively spliced isoforms that were induced by CX-4945 have not been previously reported, indicating that the effects of CX-4945 on pre-mRNA splicing involve a wide-range of abnormal alternative splicing. In addition, CX-4945 also exerted a notable effect on normal alternative splicing of Bcl-X, RON, and Clk1/sty that has been reported previously [39], [40] (Figure S2 in File S1).

This unexpected phenomenon spurred us to examine the possibility that the inhibition of CK2, the previously well-characterized target of CX-4945, may cause these changes in alternative splicing. Despite the pleiotropic functions of CK2 in diverse cellular physiologies, the direct role of CK2 in splicing has not been reported to date. Rather, it is speculative that CK2 may regulate splicing through indirect modulation of RNA metabolism, including transcription, 5′ capping, and 3′ cleavage and polyadenylation as opposed to modulation of splicing itself. Thus, it is noteworthy that CK2 physically associates with RNA polymerase II, a key component of transcription, and regulates the function of this enzyme by phosphorylation [50], [51]. In contrast to our expectations, in experiments with two other CK2 inhibitors, TBB and TBCA, changes in alternative splicing of some selected genes were not observed, although CX-4945 caused dramatic changes in the alternative splicing of these genes (Figure 3A and 3B). Consistently, siRNA-mediated knockdown of CK2 (CK2 α and/or α′) did not result in any significant changes in the alternative splicing of the selected genes (Figure S4 in File S1). Moreover, unlike CX-4945, TBB and TBCA did not induce alterations in SR protein phosphorylation (Figure S5 in File S1). Together, these results clearly demonstrate that the effect of CX-4945 on alternative splicing is not dependent on CK2 inhibition.

The CK2-independent effects of CX-4945 on splicing prompted us to identify a new target protein that is involved in splicing. Alternative splicing is regulated by the interplay between *trans*-acting factors and *cis*-acting elements on pre-mRNA [1], [10]. Particularly, SR proteins are some of the major players, and phosphorylation of these proteins is a critical event in this complex process. Several lines of evidence clearly demonstrated that CX-4945 regulates alternative splicing via modulation of SR protein phosphorylation via Clks targeting. Surprisingly, we found dramatic reductions in phosphorylation of several SR proteins (SRSF4, SRSF6, SRSF5, and SRSF1) after treatment with CX-4945, although SRSF3 was not affected. In addition, even hyperphosphorylated SRSF6 was increased markedly (Figure 4A and 4B). Similar differential effects were observed with TG-003, the well-known splicing inhibitor that selectively targets Clks, and these findings led us to determine whether CX-4945 also targets Clks as TG-003. *In vitro* kinase profiling demonstrated that CX-4945 exerts a strong inhibitory activity on all three tested Clks (Clk 1-3) with an IC_50_ of 3-90 nM (Figure 4C). In contrast, CX-4945 has relatively weak activity on SRPKs with an IC_50_ of >1000 nM, indicating the high selectivity of CX-4945 for Clks. Furthermore, the effects of CX-4945 on Clks were even greater than those of TG-003, which was the most potent inhibitor of Clks known to date (Figure S6 in File S1). Consistent with this observation, the effects of CX-4945 on the phosphorylation of SR proteins in 293T cells were found to much greater (Figure 4A). Intriguingly, of the Clks, Clk2 was most strongly affected by CX-4945, and this preferential activity has not been reported previously with any inhibitors. Indeed, most of the previously identified Clk inhibitors show a preferential effect on Clk1 and Clk4, which have similar amino acid sequences [52]. This difference in preference for Clks potentially leads to differential output with regard to the phosphorylation of SR proteins. Moreover, in order to confirm direct inhibition of Clks by CX-4945 at the cellular level, we examined the effect of CX-4945 on gluconeogenesis, which has been reported to be associated with Clk2. According to the previous report, Clk2 directly phosphorylates the SR domain of PGC-1α, resulting in transcriptional repression of gluconeogenic genes, such as G6Pase and PEPCK [53]. As anticipated, in our experiment, CX-4945 induced G6Pase and PEPCK gene expression in hepatocellular carcinoma HepG2 cells (Figure S7 in File S1). These results also support the notion that CX-4945 directly inhibits Clks in cells.

Biochemical analysis and computer-aided molecular docking studies revealed that CX-4945 acts by competing with ATP for binding to Clk2 (Figure 5). A similar mode of inhibition by CX-4945 has been previously shown for its interaction with CK2 [46]. Collectively, our results demonstrated that CX-4945 is a newly identified potent inhibitor of Clks both *in vitro* and in mammalian cells, and this new finding will increase the variety of biological tools available for splicing research.

Aberrant splicing frequently causes hereditary diseases. In fact, approximately 15% of mutations related to genetic diseases have been found to result in abnormal splicing of pre-mRNA [8]. Recently, manipulation of alternative splicing of disease-related genes using small molecules has been considered as a therapeutic approach for the treatment of splicing diseases, such as neuromuscular diseases, viral diseases, and cancers. CX-4945, which was newly characterized in this study, is anticipated to be applicable to these diseases in the future. Our findings unequivocally demonstrate a new function of CX-4945 as a potent splicing modulator and also suggest the potential use of CX-4945 for treatment of several splicing diseases.

## Supporting Information

File S1 Supporting information file containing Figures S1 to S7 and Table S1. Figure S1. Effect of CX-4945 on CK2 α′ mRNA levels. Figure S2. The effect of CX-4945 on normal alternative splicings of Bcl-X, RON, and Clk1/Sty. Figure S3. Identification of an extra PCR product of QRSL1 mRNA. Figure S4. The siRNA-mediated knockdown of the catalytic subunits of CK2, CK2α and CK2α′, does not affect alternative splicing. Figure S5. CX-4945 modulates SR protein phosphorylation in a CK2-independent manner. Figure S6. The inhibitory activities of CX-4945 on Clks were stronger than those of TG-003. Figure S7. CX-4945 induced the expression of gluconeogenic genes in HepG2 cells. Table S1. Primer sequences used in quantitative RT-PCR.
(DOCX)
