# Supplementary material for: Identification of a Novel Function of CX-4945 as a Splicing Regulator
Source: PLoS One. 2014 Apr 17;9(4):e94978. doi: 10.1371/journal.pone.0094978 (PMC3990583; doi:10.1371/journal.pone.0094978)

**Figure S1. Effect of CX-4945 on CK2 α’ mRNA levels.** Quantitative real-time PCR was performed with total RNA from DMSO- and CX-4945-treated 293T cells. The amount of normal CK2 α’ mRNA was measured using primers corresponding to exon 6, and the total amount of normal CK2 α’ mRNA and the smaller-sized CK2 α’ mRNA was measured using primers corresponding to exon 7. The values for DMSO-treated samples were set to 1, and the relative amounts were calculated. The average and SD values were determined from three independent experiments.

**Figure S2. The effect of CX-4945 on normal alternative splicings of Bcl-X, RON, and Clk1/Sty.** (A) Total RNA was prepared 12 hours after CX-4945 treatment on Huh7 cells, and RT-PCR for Bcl-X and RON was performed. (B) Total RNA was prepared 12 hours after CX-4945 treatment on 293T cells, and RT-PCR for Clk1/Sty was performed.

**Figure S3. Identification of an extra PCR product of QRSL1 mRNA**. (A) TBB induces expression of an extra QRSL1 transcript, QRSL1Δ. RT-PCR corresponding to exons 10-11-12 was performed with total RNA from DMSO- or TBB-treated 293T cells. (B) Sequence analysis of QRSL1Δ PCR product revealed that this product includes only part of exon 11 of QRSL1 mRNA (QRSL1Δexon11[113bp]: deleted of 113 bps of 5’ part of exon 11). The normally spliced (QRSL1) and alternatively spliced [QRSL1△exon11(113bp)] products are illustrated schematically.

**Figure S4. The siRNA-mediated knockdown of the catalytic subunits of CK2, CK2α and CK2α', does not affect alternative splicing.** (A) 293T cells were transfected with siRNAs against CK2α and/or CK2α'. Forty-eight hours after transfection, total extracts were prepared and subjected to western blot analysis with antibodies against CK2α and CK2α'. GAPDH was used as a loading control. (B) Total RNA was also prepared 48 hours after siRNA transfection, and RT-PCR for CK2 α, CK2 α’, ELL2, CPEB1, PRPSAP2, and QRSL1 was performed. All RT-PCRs were performed with total RNA from two independent experiments, and representative data are presented.

**Figure S5. CX-4945 modulates SR protein phosphorylation in a CK2-independent manner.** Total protein extracts from 293T cells treated with CX-4945 (10 µM), TBB (100 µM), or TBCA (100 µM) for 12 hour were separated by SDS-PAGE, and phosphorylated SR proteins were monitored by western blotting using the phosphoSR monoclonal antibody (1H4). The hnRNP A1 protein was also monitored as a control.

**Figure S6. The inhibitory activities of CX-4945 on Clks were stronger than those of TG-003.** (A) Inhibition of Clks activities by CX-4945 was measured at various concentrations using *in vitro* kinase assays conducted by Millipore. The average and SD values were determined from two independent assays*,* and IC_50_ values for each kinase were determined as described in Materials and Methods. (B) Inhibition of Clks activities by TG-003 was also measured with the same method described in panel (A), and IC_50_ values for each kinase was determined.

**Figure S7. CX-4945 induced the expression of gluconeogenic genes in HepG2 cells.** HepG2 cells were treated with CX-4945 for 12 hours, and total RNA was prepared. Quantitative real-time PCR was performed for gluconeogenic genes, PGC-1α, G6Pase, and PEPCK. GAPDH was also analyzed as a control. The average and SD values were determined from three independent experiments.


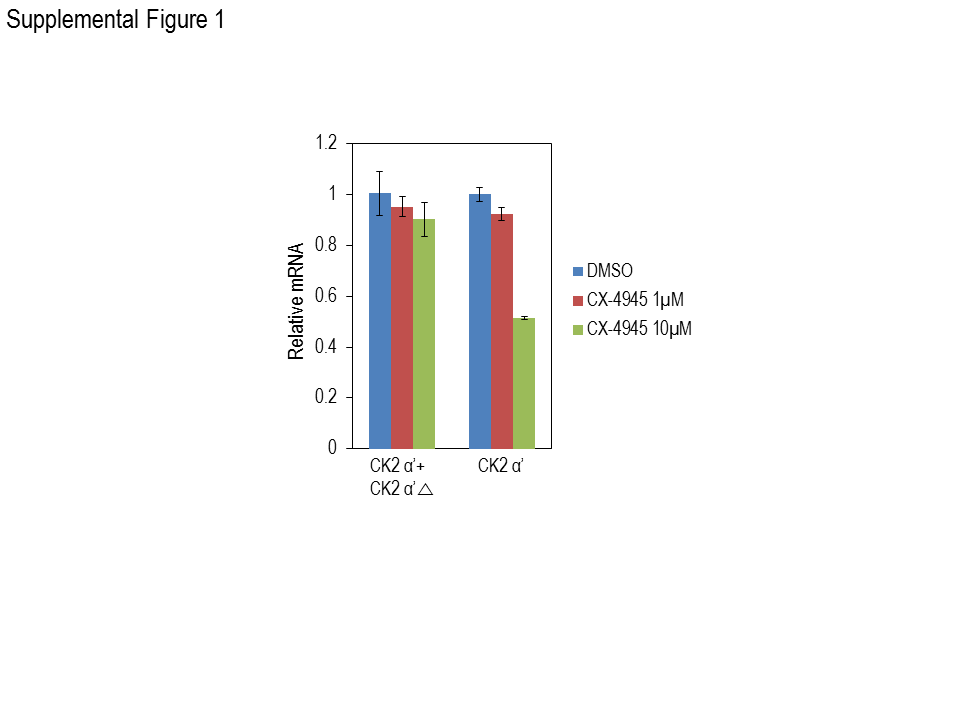

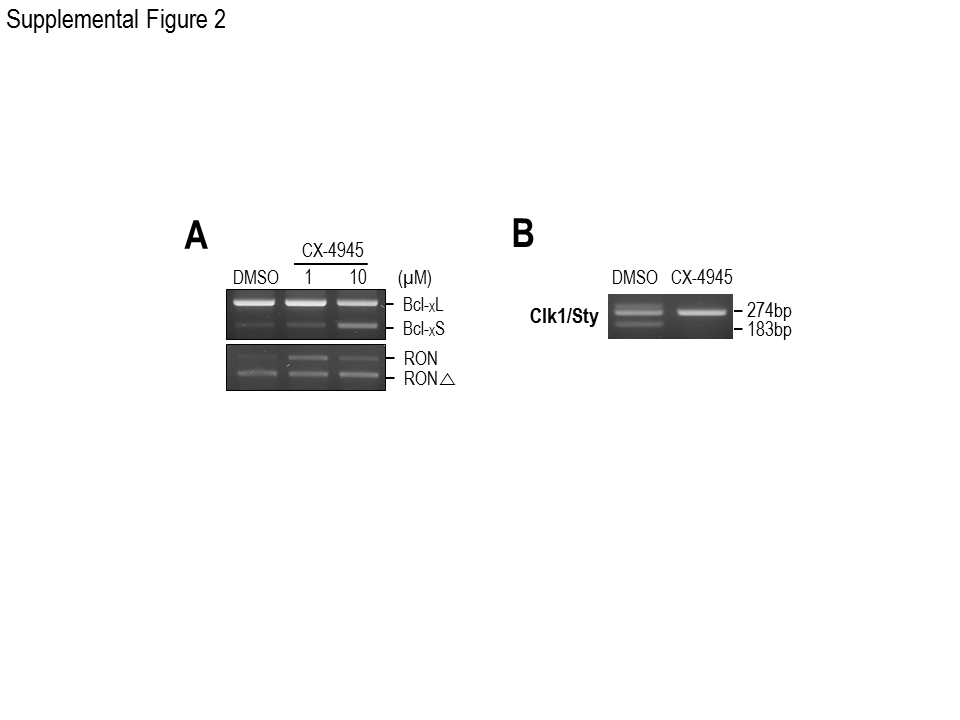

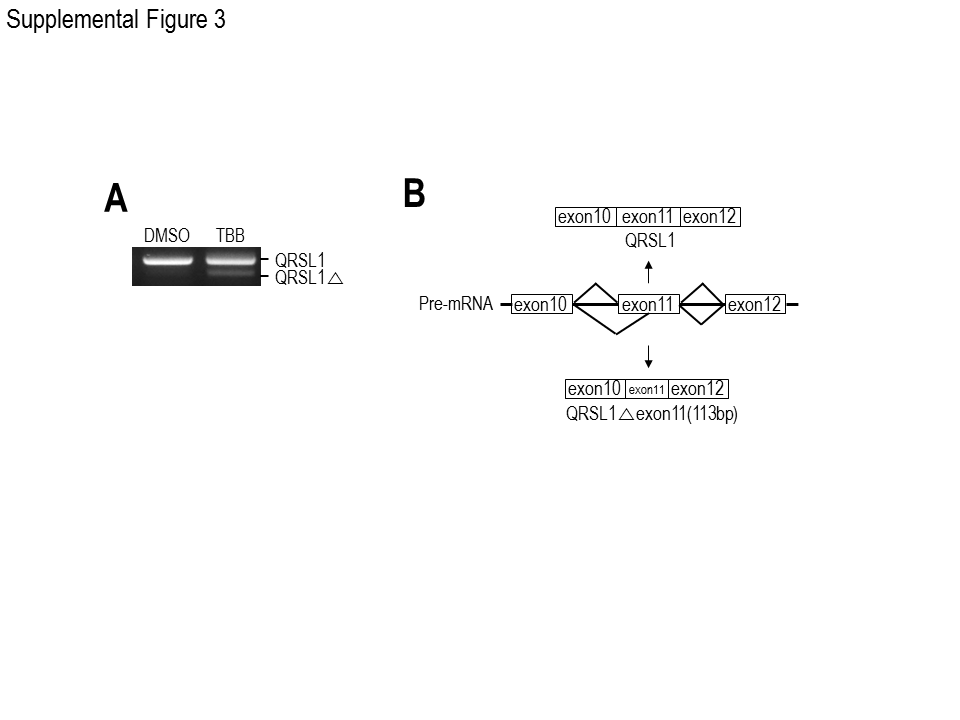

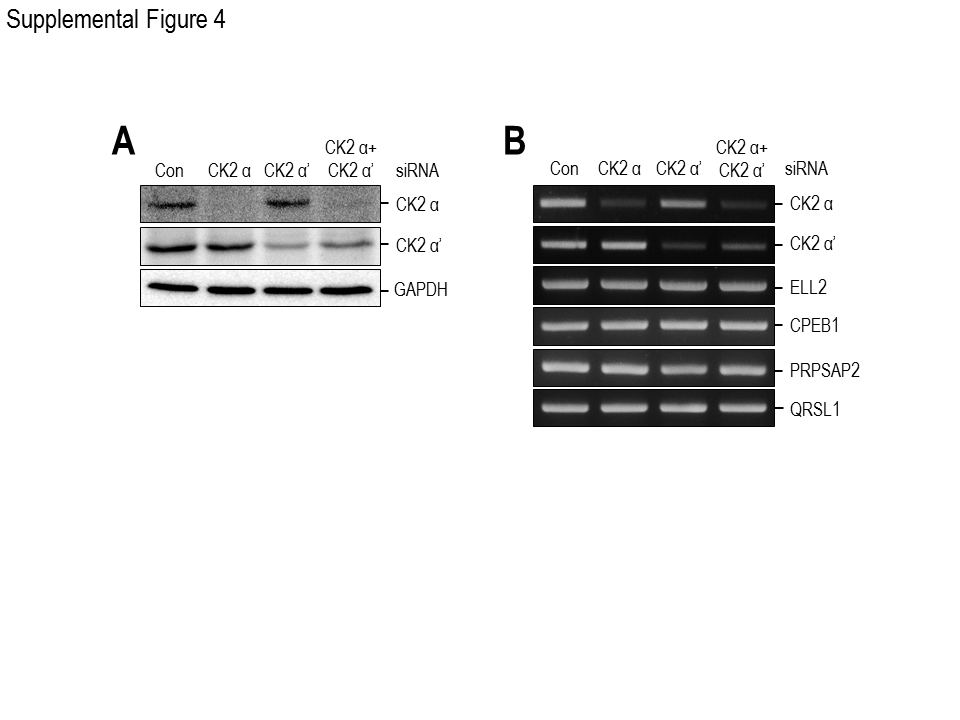

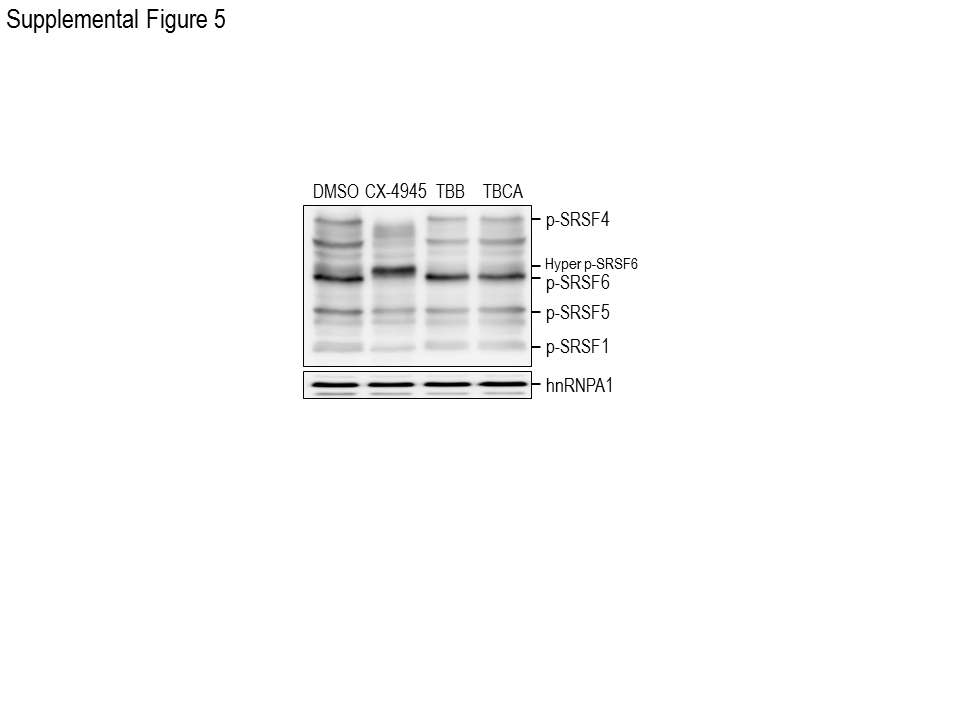

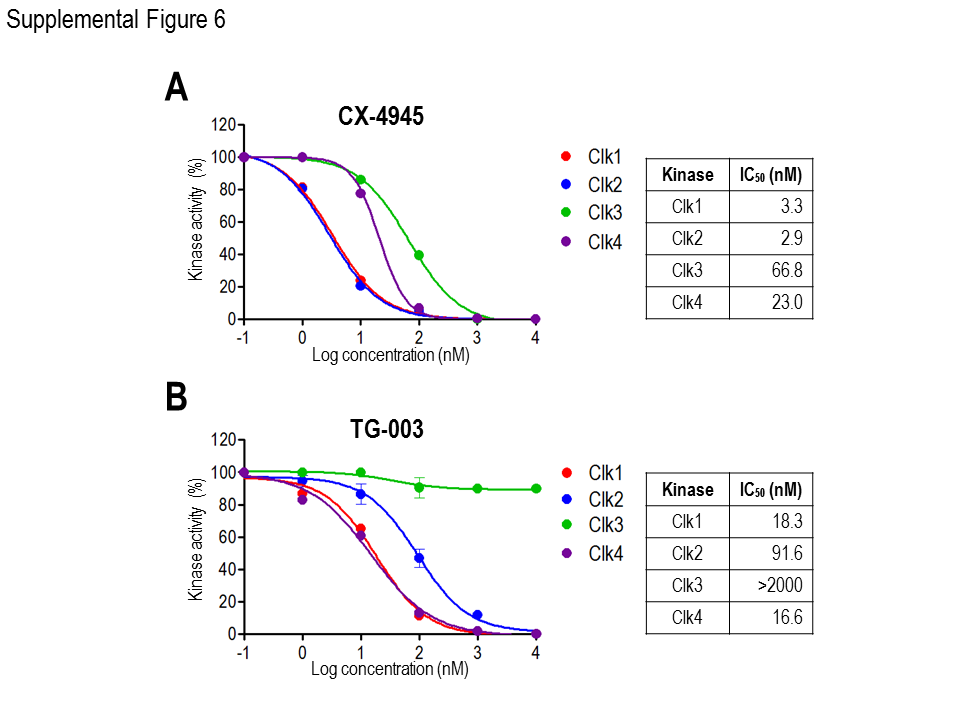

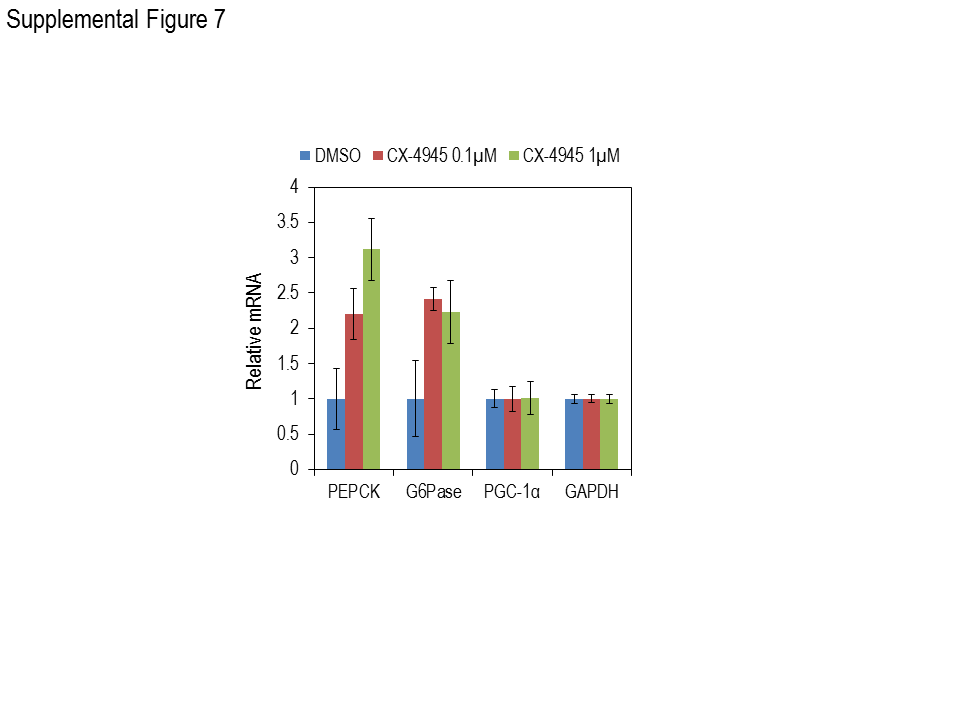

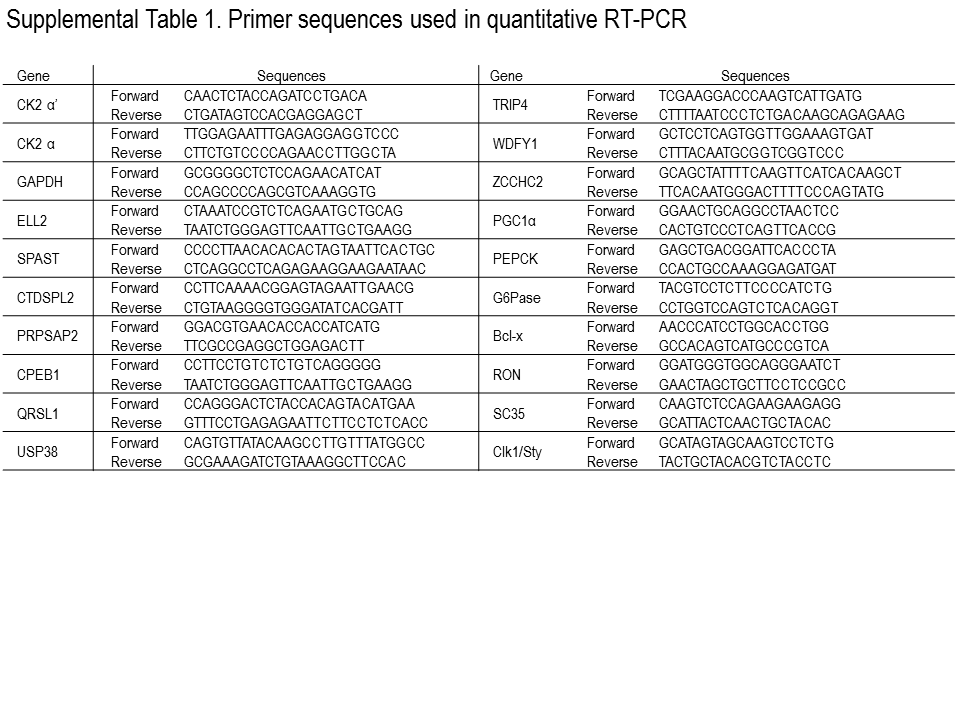

Supplement: File S1 — Supporting information file containing Figures S1 to S7 and Table S1. Figure S1. Effect of CX-4945 on CK2 α′ mRNA levels. Figure S2. The effect of CX-4945 on normal alternative splicings of Bcl-X, RON, and Clk1/Sty. Figure S3. Identification of an extra PCR product of QRSL1 mRNA. Figure S4. The siRNA-mediated knockdown of the catalytic subunits of CK2, CK2α and CK2α′, does not affect alternative splicing. Figure S5. CX-4945 modulates SR protein phosphorylation in a CK2-independent manner. Figure S6. The inhibitory activities of CX-4945 on Clks were stronger than those of TG-003. Figure S7. CX-4945 induced the expression of gluconeogenic genes in HepG2 cells. Table S1. Primer sequences used in quantitative RT-PCR. (DOCX) [file pone.0094978.s001.docx]
